# The structure and diversity of human, animal and environmental resistomes

**DOI:** 10.1186/s40168-016-0199-5

**Published:** 2016-10-07

**Authors:** Chandan Pal, Johan Bengtsson-Palme, Erik Kristiansson, D. G. Joakim Larsson

**Affiliations:** 1 Department of Infectious Diseases, Institute of Biomedicine, Sahlgrenska Academy, University of Gothenburg, SE-413 46 Gothenburg, Sweden; 2 Centre for Antibiotic Resistance Research (CARe), University of Gothenburg, Gothenburg, Sweden; 3 Department of Mathematical Sciences, Chalmers University of Technology, SE-412 96 Gothenburg, Sweden

**Keywords:** Antibiotic resistance, Biocide resistance, Metal resistance, Resistome, Metagenomics, Human microbiome, Environmental microbiome

## Abstract

**Background:**

Antibiotic resistance genes (ARGs) are widespread but cause problems only when present in pathogens. Environments where selection and transmission of antibiotic resistance frequently take place are likely to be characterized by high abundance and diversity of horizontally transferable ARGs. Large-scale quantitative data on ARGs is, however, lacking for most types of environments, including humans and animals, as is data on resistance genes to potential co-selective agents, such as biocides and metals. This paucity prevents efficient identification of risk environments.

**Results:**

We provide a comprehensive characterization of resistance genes, mobile genetic elements (MGEs) and bacterial taxonomic compositions for 864 metagenomes from humans (*n* = 350), animals (*n* = 145) and external environments (*n* = 369), all deeply sequenced using Illumina technology. Environment types showed clear differences in both resistance profiles and bacterial community compositions. Human and animal microbial communities were characterized by limited taxonomic diversity and low abundance and diversity of biocide/metal resistance genes and MGEs but a relatively high abundance of ARGs. In contrast, external environments showed consistently high taxonomic diversity which in turn was linked to high diversity of both biocide/metal resistance genes and MGEs. Water, sediment and soil generally carried low relative abundance and few varieties of known ARGs, whereas wastewater/sludge was on par with the human gut. The environments with the largest relative abundance and/or diversity of ARGs, including genes encoding resistance to last resort antibiotics, were those subjected to industrial antibiotic pollution and a limited set of deeply sequenced air samples from a Beijing smog event.

**Conclusions:**

Our study identifies air and antibiotic-polluted environments as under-investigated transmission routes and reservoirs for antibiotic resistance. The high taxonomic and genetic diversity of external environments supports the hypothesis that these also form vast sources of unknown resistance genes, with potential to be transferred to pathogens in the future.

**Electronic supplementary material:**

The online version of this article (doi:10.1186/s40168-016-0199-5) contains supplementary material, which is available to authorized users.

## Background

Accelerating antibiotic resistance development in pathogens is a major threat to modern health care [1] and has been estimated to cause more than 700,000 deaths yearly [2]. This development has to a large extent been enabled by the recruitment of antibiotic resistance genes (ARGs) into bacterial pathogens via mobile genetic elements (MGEs) such as integrons, transposons and plasmids [3]. Going back to the pre-antibiotic era, plasmids were mostly devoid of ARGs [4, 5]. Similarly, bacteria isolated from wild animals in remote areas with no history of antibiotic exposure rarely carried ARGs [6, 7]. However, the use and abuse of antibiotics have increased the prevalence of resistance genes in the human and animal microbiome over the last 75 years [8]. Since the 1940s, significant increases of ARGs have also been reported in farmland soils [9, 10]. The transfer of ARGs between bacteria of human and animal origins has also been documented, and resistant bacteria in the animal microbiome can thus serve as reservoir of clinically important ARGs [11].

In the environment, resistance is ancient. Several ARGs and genes similar to known ARGs can be found in remote environments with minimal anthropogenic impact across the globe, such as 30,000-year-old permafrost, isolated caves, Alaskan soil and glaciers [12–15]. There are also other lines of evidence suggesting that many, perhaps the majority, of the ARGs found in pathogens today, have an environmental origin [16–18]. This clearly emphasizes the importance of environmental bacteria as potential sources for clinically important forms of resistance. Despite this, environmental resistomes are still largely unexplored and little attention has been paid to their intrinsic capacity to retain and transfer resistance. Surveillance programs on antibiotic usage and resistance characteristics of bacterial pathogens are in place in different parts of the world [1]. Corresponding environmental monitoring programmes are yet in their infancy, although the need has been identified [19, 20].

Understanding of the environment as a source and dissemination route for ARGs is fundamental in order to identify risk scenarios for human health [21]. In this context, both the abundance and diversity of resistance genes need to be considered. Environments with a large diversity of resistance genes not generally present in the human microbiome are potential sources for recruitment of ARGs to pathogens [22]. High abundances of resistance genes in a particular environment may also reflect selection for resistance determinants, directly or indirectly, in that environment. Alternatively, it may reflect contamination with antibiotic-resistant bacteria and hence risks for their transmission [23]. That said, the diversity of any type of genes, including ARGs, is likely to be associated with taxonomic diversity of the community. Finally, fast turnaround of ARGs and taxa in an environment suggests certain robustness to perturbations through establishment of such transient ARGs and taxa under favourable conditions, such as antibiotics exposure [24].

Shared ecological adaptations among bacteria are known to be important for the ability to engage in horizontal gene transfer [25]. Defining specific bacterial taxa that thrive in several different habitats is therefore important, as they may serve as mediators for ARGs in crossing ecological dispersal barriers. However, to comprehend the flow of ARGs between environments, identifying ARGs that are widespread in both the human microbiome and other habitats is important. These are also less likely to contribute substantially to future health risks associated with novel recruitments of resistance determinants from the environment [22].

Antibacterial biocides and metals may also contribute to the promotion of antibiotic resistance via co-selection [26]. Resistance genes to such compounds are occasionally co-located with ARGs on MGEs such as plasmids, integrons and transposons [27], which enable their transfer within bacterial cells, between bacterial species and between environments with sufficient ecological connectivity. However, the abundance and diversity of MGEs and resistance genes to biocides and metals in environmental, animal and human microbial communities are poorly investigated to date. Tracking of ARGs, MGEs and resistance genes to potentially co-selective agents across human, animal and external environments can contribute to the understanding of the ecology and epidemiology of antibiotic resistance and uncover the relevance of environmental bacteria in the spread and transfer of antibiotic resistance to humans.

Earlier efforts have estimated the distribution and relative abundance of ARGs across multiple environments using 32 [28] and 71 [29] metagenomic datasets generated on 454 and Sanger sequencing platforms. Such small sample sizes, low sequencing depth and/or non-stringent criteria for resistance gene detection make it difficult to generalize results. Recently, Fitzpatrick and Walsh [30] reported the distribution and relative abundance of ARGs across environments using 432 metagenomic datasets with highly variable sequencing depths generated by a range of different sequencing platforms. However, comparisons of datasets generated on different sequencing platforms is not trivial as properties such as total read number, base pair volume, and average read length produced by different sequencing platforms vary substantially and indirectly influence the abundance counts, making comparisons ambiguous [29].

In contrast, we have in this study characterized broad types of environments with regard to abundance and diversity of resistance genes to antibiotics, biocides and metals, as well as markers of MGEs, with the ultimate aim to identify environments which could act as transmission routes and sources for ARGs to pathogens. To achieve this, we have also identified similarities and differences of antibiotic resistomes and bacterial taxa distributions between environments. To allow a comprehensive and comparable analysis, we conducted a large-scale metagenomic survey and quantitative comparison using 864 deeply sequenced metagenomes, all generated on Illumina sequencing platforms, from humans, animals and a range of external environments.

## Results

### The abundance and richness of ARGs across environments

**Fig. 1:**
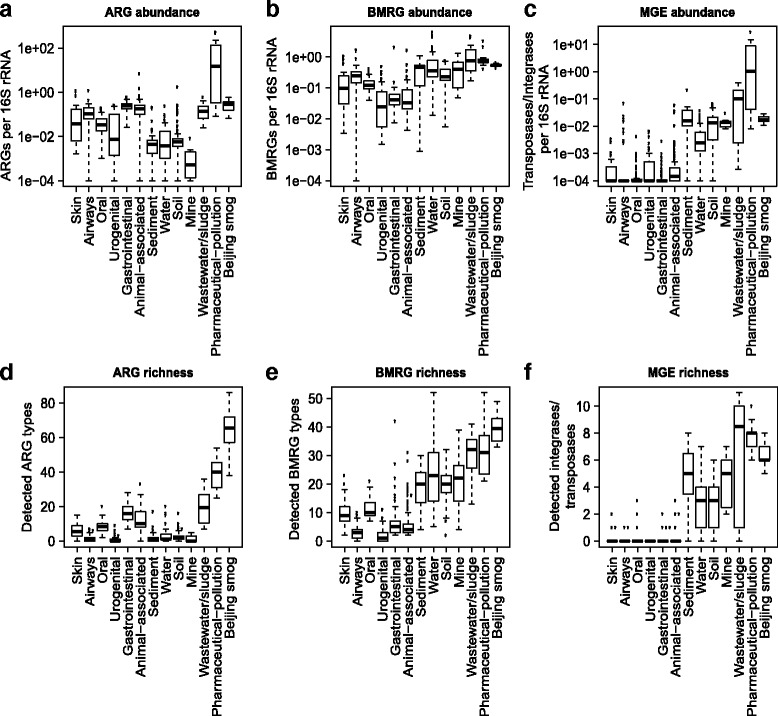
Relative abundance and richness of resistance genes to antibiotics, biocides and metals, as well as mobile genetic elements across environments. The *upper row* shows the relative abundance of **a** antibiotic resistance genes (ARGs), **b** biocide/metal resistance genes (BMRGs) and **c** mobile genetic elements (MGEs). The *plots* in the *second row* show the richness of **d** antibiotic resistance genes, **e** biocide/metal resistance genes and **f** mobile genetic elements. The relative abundance and richness are presented with the median (*central black horizontal line*); 25th and 75th percentiles (*box*) and whiskers extend from each end of the box to the most extreme values within 1.5 times the interquartile range from the respective end. Whiskers data points beyond this range are displayed as *small black circles *

The presence and relative abundance of 325 known ARG types, 131 known biocide/metal resistance gene types and 17 known MGEs were investigated in 864 metagenomes. The median relative abundance of ARGs was 0.035 copies per 16S ribosomal RNA (rRNA). The median richness, calculated as the total number of unique resistance genes per 10 million reads, was 5. The relative abundance and richness showed large variability, both between and within environments (Fig. 1).

### Antibiotic-polluted environments have the highest abundances of ARGs

Environments affected by pollution from pharmaceutical manufacturing were not only rich in ARGs but also carried the highest relative abundance of ARGs of all investigated environments (Fig. 1a). In particular, we identified exceptionally high relative abundances of the sulfonamide resistance gene *sul*2 and aminoglycoside resistance genes *aph*(6)*-Id* and *aph*(3”)*-Ib* together with a set of resistance genes to quinolones (*qnr*) and beta-lactams (Additional file 1: Figure S1). The exact quantitative estimates of resistance genes should, however, be interpreted with caution since DNA from 7 out of 11 metagenomes from pharmaceutically polluted environments were amplified before sequencing, which can potentially introduce bias. The relative abundances of ARGs in wastewater/sludge were also higher (0.17 copies per 16S rRNA on average) compared to most other environmental habitats (sediment, water, soil and mine; 0.002–0.02 copies per 16S rRNA on average) all of which are likely less impacted by human faecal residues. Air from Beijing smog (see below), pharmaceutically polluted (38.9 different ARG types) and wastewater/sludge (19.4 different ARG types) environments carried more diverse sets of ARGs than did metagenomes from other external environments (1.6–3.3 different types of ARGs), animals (11.8 different ARG types) and humans (1.0–16.6 different ARG types) (Fig. 1d). Note that these estimates only refer to genes identical or highly similar to known ARGs.

### Urban air has high abundance and diversity of ARGs

**Fig. 2:**
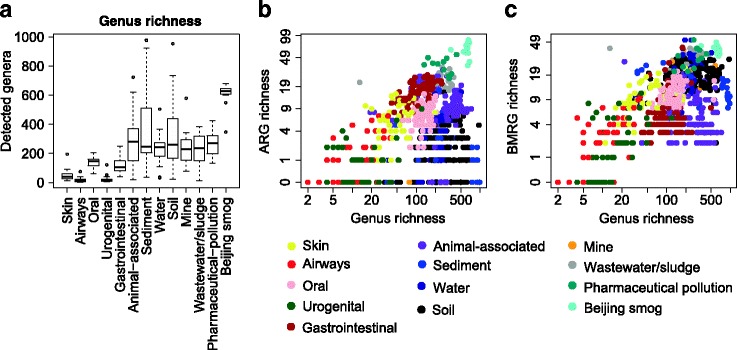
Diversity (richness) of taxa and relationship between richness of bacterial genera and resistance genes expressed per 10 million reads. **a** Richness of bacterial genera is presented with the median (*central black horizontal line*); 25th and 75th percentiles (*box*) and whiskers extending from each end of the box to the most extreme values within 1.5 times the interquartile range from the respective end. Whiskers data points beyond this range are displayed as *small black circles*. **b** Genus richness versus richness of antibiotic resistance genes (Spearman’s correlation coefficient = 0.073, *p* = 0.0319). **c** Genus richness versus richness of biocide/metal resistance genes (Spearman’s correlation coefficient = 0.462, *p* < 0.001). Richness values were log-transformed before performing Spearman’s rank correlation. A value of 1 was added to the richness matrix to avoid zeros in log-transformed richness values in the correlation test

Microbial communities from Beijing smog harboured the highest richness of known ARGs (64.4 different ARG types), as well as the highest bacterial richness of all environments (Fig. 2a). The relative abundance was however on the same level as the human gut and wastewater/sludge (0.3 copies of ARGs per 16S rRNA). To investigate if the high ARG richness was a general feature of air microbiomes, we compared the resistome profile of Beijing smog samples to indoor and outdoor air samples (generated on the 454 sequencing platform) from houses, office buildings and hospitals located in New York and San Diego [31]. After normalizing for the very large differences in sequencing depth between the two datasets (using down-sampling), the air microbiomes from the US cities showed comparable relative abundances of ARGs. However, the richness of ARGs was higher in Beijing smog than in the air samples from US cities with the exception of office indoor air samples (Additional file 1: Figure S2). Notably, the Beijing smog metagenomes contained several resistance genes to carbapenems, a class of last resort antibiotics, including IND, GES, IMP, OXA-50, OXA-51 and OXA-58 beta-lactamase gene types (Additional file 2: Table S1).

### Human microbiota has high abundance and diversity of ARGs but low taxonomic diversity

The human microbiomes carried on average higher relative abundances of ARGs than most of the investigated external environments, with the exceptions of wastewater/sludge, pharmaceutically polluted environments and Beijing smog. Within human body sites, relative abundances and richness of ARGs were highest in the gut (Fig. 1a, d). Notably, the human gut also contained a higher ARG richness (16.6 different ARG types) compared to samples from animal sources (11.8 different ARG types), but their relative abundance level was similar (0.26 copies ARGs per 16S rRNA). Taxonomic richness, measured as the unique number of genera per 10 million reads, was consistently low in human microbiota (Fig. 2a). When looking at all types of environments together, the ARG and taxonomic richness showed a weak correlation (Fig. 2b; Spearman’s correlation coefficient = 0.073, *p* = 0.0319).

### Tetracycline resistance dominates human and animal microbiomes

**Fig. 3:**
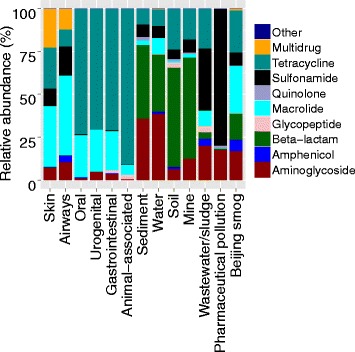
*Bar graph* showing the relative abundance of resistance genes to different classes of antibiotics across environments

We also analysed the abundance distribution of the classes of ARGs across humans, animals and external environments (Fig. 3). Overall, genes providing resistance to tetracycline were the most common type of ARGs across environments. The human gut, oral and urogenital antibiotic resistomes were dominated by resistance genes to tetracyclines and macrolides, while the resistome of skin and airways had a wide distribution of resistance gene classes. Notably, over 90 % of the resistance genes identified in animal-associated metagenomes provided resistance to tetracyclines. Furthermore, in contrast to human and animal sources, external environments harboured much higher relative abundances of beta-lactam resistance genes. Wastewater/sludge and pharmaceutically polluted environments had higher relative abundances of sulfonamide resistance genes than other types of environments did. Notably, more than 99 % of the ARGs from pharmaceutically polluted environments provided resistance to sulfonamides, aminoglycosides and quinolones, but behind this dominance, a large diversity of ARGs was still present. Urban air from Beijing harboured a comparatively even distribution of resistance genes to different classes of antibiotics.

### Many ARGs are widespread across environments

Out of the 325 horizontally transferable ARG types analysed, 203 ARG types were detected at least once in this study (Additional file 3: Table S2). Out of these 203 ARG types, 12 (6 % of the detected ARGs) were found in at least 9 out of 13 investigated environments and could therefore be considered ‘widespread’. These included resistance genes to aminoglycosides [*ant*(3”)*-Ia*, *aph*(3’)*-Ib*, *aph*(3’)*-IIa* and *aph*(6)*-Id*], macrolides [*erm*(B)], beta-lactams (TEM) and tetracyclines [*tet*(32), *tet*(M), *tet*(O), *tet*(Q), *tet*(W) and *tet*(X)]. Furthermore, genes widespread in the human microbiome (four out of five body sites) included the aminoglycoside resistance genes [*aph*(3’)*-Ia*, *aph*(3’)*-Ib* and *aph*(6)*-Id*], tetracycline resistance genes [*(tet*(Q), *tet*(W), *tet*(O), *tet(*M), *tet*(32) and *tet*(37)] and macrolide resistance genes [*erm*(B) and *erm*(X)] (Additional file 3: Table S2). Similarly, a set of resistance genes to aminoglycosides [*ant*(2”)*-Ia*, *ant*(3”)*-Ia*, *aph*(3”)*-Ib* and *aph*(3”)*-IIa*], beta-lactams (TEM and CMY2), quinolones (qepA), tetracyclines [*tet*(32), *tet*(C), *tet*(O), *tet*(W), t*et*(X) and *tetB*(P)] and macrolides [*vat*(F)] were widespread (detected in five out of seven) across external environments. Over half of the ARGs were only detected in external environments (57.5 %), while 20.5 % were found in human, animal and at least one of the external environments (Additional file 1: Figure S3). Interestingly, only 4.5 % of the ARGs were only found in the microbiomes of animals and/or humans. However, 3.5 % of all detected ARGs were found both in animals and at least one of the external environments, whereas 14 % of the detected ARGs were found in both humans and at least one external environment.

### Biocide and metal resistance genes are most common in external environments

The relative abundances of biocide and metal resistance genes were, in contrast to ARGs, higher in most external environments than in human and animal microbiomes (Fig. 1b). Similarly, the richness of biocide and metal resistance genes was higher in all investigated external environments than in human body sites and animals, with Beijing smog having the highest richness of biocide and metal resistance genes (Fig. 1e). Within the human microbiome, oral and skin habitats showed higher richness of biocide/metal resistance genes than other body sites did. There was a strong correlation between the richness of biocide/metal resistance genes and the genus richness (Spearman’s correlation coefficient = 0.469, *p* < 0.001) (Fig. 2c). We observed no correlation between the richness of ARGs and biocide/metal resistance genes (Spearman’s correlation coefficient = −0.015, *p* = 0.645), even after controlling for the effect of taxonomic richness (partial correlation coefficient = −0.056, *p* = 0.097) (Additional file 1: Figure S4).

### Human microbiota carries low abundance and richness of MGEs

The relative abundances of known MGEs were found to be highest in environments polluted by discharges from pharmaceutical production and in wastewater/sludge (Fig. 1c). In contrast, human and animal microbiomes carried much lower abundances of MGEs. Similarly, the richness of MGEs was found highest in pharmaceutically polluted environments and wastewater/sludge and lowest in humans and animals (Fig. 1f). Notably, the MGE richness was especially low in the human microbiome, which was dominated by the transposases ISCR2, ISCR5 and ISCR8 and integron-associated integrase class 1 (*intI1*). However, some other classes of ISCRs, such as class of 1, 4, 6, 7 and 14, and most classes of integrases (except *intI1*), were found in very low frequencies (Additional file 1: Figure S5). This was in contrast to the external environments where almost all types of investigated, known MGEs were detected in relatively high abundances.

### Resistance profiles and taxonomic compositions are structured by environment

**Fig. 4:**
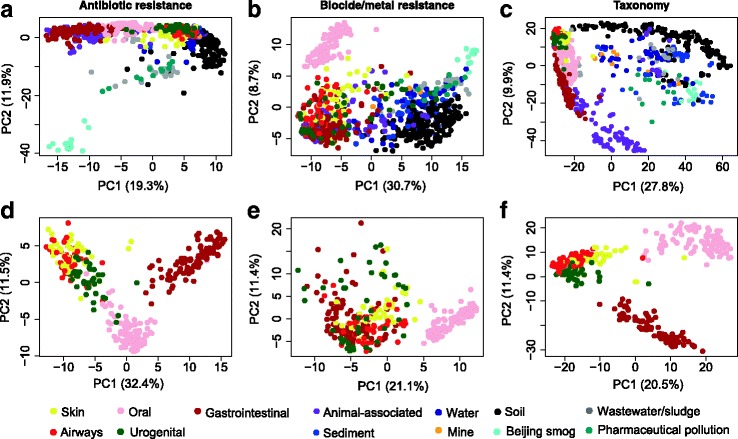
Principal component analysis of resistance genes and bacterial genera. The *upper row* shows the variation of **a** antibiotic resistance genes, **b** biocide/metal resistance genes and **c** bacterial taxa (genus level) among samples from all investigated environments, including human body sites and animals. The *plots* in the *second row* show the variation of **d** antibiotic resistance genes, **e** biocide/metal resistance genes and **f** bacterial genera among samples from the human microbiota only

The resistomes and the taxonomic compositions of the different environments were further analysed using principal component analyses (Fig. 4). Most investigated environmental, human and animal samples clustered distinctly but with some overlap. Samples from similar environments mostly clustered together independently of their geographical locations. Soil samples showed a higher degree of variation than did samples from other environments. In terms of ARG profiles, human and animal samples clustered together (Fig. 4a). Similarly, ARG profiles of wastewater/sludge and environments with pharmaceutical pollution clustered together while Beijing smog had distinctly isolated profiles. Within humans, the ARG profiles of gastrointestinal, oral and urogenital samples separated from each other, whereas skin and airways samples clustered together but separately from other habitats (Fig. 4d). For biocide and metal resistance gene profiles, oral samples formed an isolated cluster while samples from external environments clustered separately from humans and animals (Fig. 4b, e). However, samples from humans and animals overlapped extensively.

Similar to the biocide and metal resistance gene profiles, the taxonomic profiles of human and animal samples partially overlapped but were separated from environmental samples (Fig. 4c). In contrast, human body sites were clustered by habitat. Notably oral and gastrointestinal samples were separated from skin, airways and urogenital samples (Fig. 4f).

### Beta-diversity of resistance genes and taxa

**Fig. 5:**
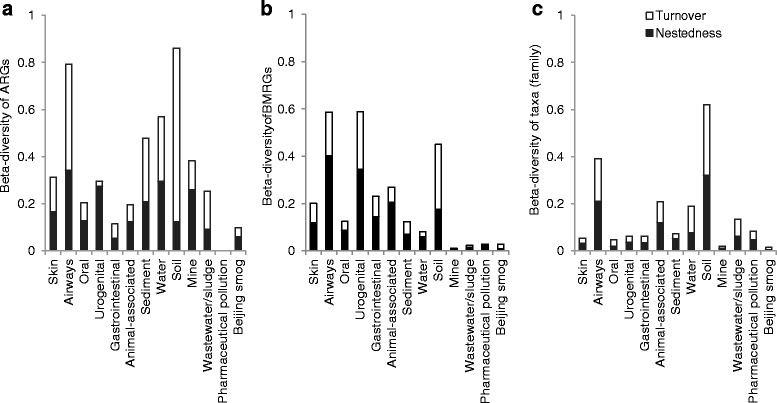
Beta-diversity of resistance genes and taxa. The figure showing beta-diversity of **a** antibiotic resistance genes (ARGs), **b** biocide/metal resistance genes (BMRGs) and **c** bacterial taxa (family level) across environments. Beta-diversity is expressed as the Sørensen-based multiple-site dissimilarity and is further partitioned into turnover and nestedness

The between-sample diversity (i.e. beta-diversity) of both ARGs and taxa differed between environments, but there was no consistent difference between human and external environments (Fig. 5; Additional file 4: Table S3). In contrast, the beta-diversity of biocide and metal resistance genes was lower in external environments, except for soils. Furthermore, soil had a higher beta-diversity of taxa than all other environments. The low beta-diversity of both pharmaceutically polluted environments and Beijing smog reflects that the same, diverse set of ARG were present across all samples, which for both environment types came from a limited geographical region.

Beta-diversity was further partitioned into ‘turnover’ (i.e. replacement of genes or taxa between samples) and ‘nestedness’ (i.e. loss of nested genes or taxa between samples) components [32]. For ARGs, turnover explained the most of the beta-diversity in soil, sediment, wastewater/sludge and airways (Fig. 5). Turnover also explained most of the biocide and metal resistance gene beta-diversity in soil. In contrast, for most of the human and animal microbiomes, nestedness contributed more to beta-diversity than turnover. This suggests that the resistome varies across soil and wastewater/sludge microbial communities due to the presence of new individual resistance genes in each additional sample taken from the same environment type, whereas a larger set of resistance genes are shared between samples from human and animal microbiomes.

### Resistome and taxonomic similarity between environments

**Fig. 6:**
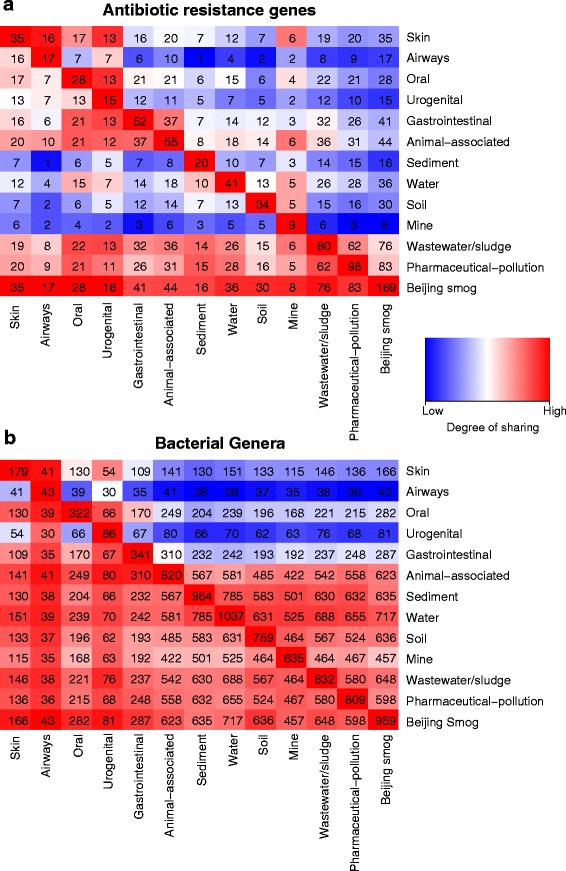
Shared resistance genes and taxa between environments. Each number in the co-ordinate grid shows the absolute number of **a** antibiotic resistance genes or **b** bacterial genera that are shared between the environments listed on the *horizontal axis* and the environments listed along the *vertical axis*. The *colour scale* reflects the degree of sharing (percent of resistance genes or taxa from the environment on the horizontal axis present in the environment on the vertical axis)

Though ARGs detected in the human gut were often shared with animals (71 %, 37 ARGs) and wastewater/sludge (62 %, 32 ARGs), just over 40 % of ARGs detected in wastewater/sludge were shared with the human gut (32 ARGs) and animals (36 ARGs) (Fig. 6a). Moreover, soil, water and sediment microbial communities shared much lower numbers of ARGs with the human microbiome than with other investigated habitats. For example, around 25 % of ARGs detected in the human gut microbiome was shared with soil (12 ARGs) and water (14 ARGs) microbiomes.

Large numbers of bacterial genera (48–84 % of total detected genera) were shared between external environments (Fig. 6b). In contrast, only 31 % of the total detected genera were shared between environmental and human microbiotas (Additional file 1: Figure S3). The taxonomic composition of the human microbiota largely resembled that described previously [33]. Interestingly, only 28.5 % of the genera found in wastewater/sludge were shared with the human gut (237 genera), whereas 65 % of genera found in wastewater/sludge were shared with animals (542 genera) (Fig. 6b). Two thirds of the genera found in the human gut or animals were shared with wastewater/sludge. It should be noted that a large proportion of bacteria from external environments (on average, 63.9 %) and animals (56.3 %) could not be classified even to the genus level (Additional file 1: Figure S6 and S7).

## Discussion

To the best of our knowledge, this is the most comprehensive characterization to date of antibiotic, metal and biocide resistomes, as well as markers of MGEs, covering human, animal and external environments. Environments polluted with discharges from pharmaceutical production and Beijing smog carried the largest relative abundance and diversity of ARGs, followed by wastewater/sludge, human and animal microbiomes with intermediate figures, and considerable lower counts in other external environments. The explanation behind the high relative abundance of ARGs in pharmaceutically polluted environments is most likely an exceptionally strong, prevailing antibiotic selection pressure, whereas the high diversity of resistance genes, taxa and MGEs found in smog is more likely a consequence of air coming into contact with many different environments with different types of bacteria. Conceivably, depending on the host bacterium, ARGs could have other functions that are not directly related antibiotic resistance, which could contribute to their abundance in different environments [34, 35]. Regardless of the causes, our observations suggest that urban air and pharmaceutically polluted environments warrant further investigation of their roles as reservoirs and point sources of ARGs. Previous meta-analyses of the diversity of ARGs in metagenomes from different environment types have not included air and pharmaceutically polluted environments. Hence, their potential importance has largely gone unobserved [28–30]. Human and animal microbiomes stood out by having the lowest relative abundance and diversity of both MGEs and biocide/metal resistance genes. This may, at least partially, be explained by lower taxonomic diversity in these communities. The much larger taxonomic diversity, together with a multitude of mechanisms for genetic mobility and larger beta-diversity of ARGs in external environments, supports the hypothesis that these form vast sources of unknown resistance genes, with potential to be transferred to pathogens in the future.

Recurring smog events in Beijing and other megacities are already growing public health issues [36, 37]. We interpret the high relative abundances of ARGs in smog as a reflection of that air comes into direct contact with many other types of environments, thereby accumulates a highly diverse collection of bacteria, including resistant ones. This is further supported by a very high taxonomic richness in these samples. We are not aware of any other deeply sequenced shotgun data from air, preventing us from generalizing our results to other air environments. When we investigated metagenomes of air samples from the USA, albeit produced by 454 technology to a considerably smaller sequencing depth, we found preliminary support for similarly large relative abundances of ARGs. However, the diversity in the US air samples was overall lower than in Beijing smog. Using culture-based approaches, air samples from wastewater treatment plants, animal slaughter houses and swine feeding operations have earlier been shown to carry resistant bacteria [38–40]. In addition, aerial transport of antibiotic-resistant bacteria from cattle feed yards was recently proposed [41], but in general, the primary sources and the importance of resistant bacteria in urban air are not clear. Taken together, this highlights that air transmission is, to this point, an under-investigated route for the spread of resistance. Although we do not know the proportion of live bacteria in smog, neither the bacterial hosts of the ARGs, we think that the finding of resistance genes such as IND, GES, IMP, OXA-50, OXA-51 and OXA-58 carbapenemases calls for concern given the growing global threat of carbapenem-resistant Enterobacteriaceae [42, 43]. That said, the Beijing smog samples were collected from a single smog event that lasted for 5 days (January 10–14, 2013) [36], and thus, air samples from more types of environments with different potential sources of bacteria taken at other locations would provide a clearer picture regarding air as a dissemination route of antibiotic resistance.

It seems unlikely that antibiotic selection is an important factor shaping the air resistome, given the limitations for most bacteria to grow in air. In other environments, the ARG profiles may be more influenced by direct selection from antibiotics or other chemicals. This includes environments polluted by wastewater from the manufacturing of antibiotics [44]. Recent culture-dependent and culture-independent studies suggest that these indeed are the most extreme environments described to date on earth, not only when it comes to multi-resistance to antibiotics but also in terms of carrying integrons of classes 1 and 2, known to often be associated with ARGs [45–47]. Bacteria from a polluted lake had the ability to transfer novel resistance plasmids to *E. coli*, stressing the potential role of industrial antibiotic pollution in the emergence of resistance in pathogens [48]. This raises strong concerns about the risks for human health associated with discharges of high levels of antibiotics and warrants both actions to reduce discharges [49] and deeper investigation of the role of pharmaceutical pollution in the emergence and transmission of resistance.

Humans and domesticated animals are regularly exposed to selective concentrations of antibiotics during therapy, inevitably driving resistance. Accordingly, the strong dominance of tetracycline resistance genes in the animal microbiomes, also identified by Durso et al. [28], may partially be explained by current and historical exposure to selective concentrations of tetracyclines, as this is the most commonly used antibiotic class for animals worldwide [50, 51]. Whereas tetracycline is known to promote enrichment of *tet*-genes in communities to a larger extent than it promotes other classes of ARGs [52], co-selection between classes may also be important. Thus, based on the type of ARGs found, it is not straightforward to conclude which classes of antibiotics that could have provided a selection pressure. Background knowledge of resistance genes that usually occur in a given environment type allows identification of deviations from the norm. Therefore, overrepresentation of resistance genes could provide clues to what selective agents that could be present. In environments other than human, animal and pharmaceutically polluted ones, it is considerably less clear if the levels of antibiotics, or for that sake also metals and biocides, are sufficiently high to select for ARGs. Relatively strong correlation between richness of genera and biocide/metal resistance genes does not support a role of environmental-specific selection pressures, but does of course not exclude that it occurs in individual cases. Note that the correlation with taxonomic diversity is considerably lower for (known) ARGs, as ARG diversity can be low despite very large taxonomic diversity. In contrast to ARGs, relative abundance and diversity of biocide/metal resistance genes were higher in environmental microbiomes than in the human microbiome, which is highly consistent with our previous study of antibiotic, biocide and metal resistance genes on plasmids from bacterial isolates of multiple environments [27]. Analyses of metagenomes as performed here, compared to studies of isolates, allow insight also into the uncultivable portion of different communities but pays the price of not providing the genetic context of resistance genes. Short-read metagenomic assembly approaches for determining the genetic context of resistance genes in complex metagenomes still face many technical limitations mainly because resistance genes occur in multiple contexts [47, 53]. The recently developed epicPCR methodology has the potential to address at least some of these shortcomings of metagenomics [54].

The variable abundances and types of resistance genes are only partially governed by the selection pressure within each environment type. Transmission between environments, primarily from human and animal sources, which typically carry larger relative abundances of ARGs than most external environments, are likely to play an important role as well [55]. This is a particularly tenable explanation for the relatively high abundance and diversity of ARGs in sewage/sludge. In this type of environment, the complex mixtures of antibiotics, detergents and metals could also, if present at sufficiently high levels, provide a selection pressure for antibiotic-resistant bacteria [56], but clear-cut evidence for this is still lacking [57]. Some of the high relative abundance and diversity of ARGs in the human microbiota is very likely a consequence of transmission between humans rather than a direct effect of antibiotic selection pressure in the studied individuals. This interpretation does not only fit the general view of how humans tend to share microbiota with each other but also supported in our analysis by the comparably low and highly nested beta-diversity of ARGs found in the human microbiota (except airways) compared to, e.g. soil. Evidence for the role of transmission in determining the ARG profiles of human-associated bacteria can easily be seen on larger geographical scales, where we find dominance of certain ARGs in human pathogens from some regions, while other genes with similar resistance function dominate in other countries, for example, KPC carbapenemases in the USA and NDM-type carbapenemases in India [58, 59].

In addition to selective pressures and transmission, the different biotic and abiotic conditions associated with different environments also favour certain types of bacteria, indirectly favouring resistance genes that tend to be associated with those taxa. Data on taxonomic composition may therefore provide possible explanations to the overrepresentation of specific resistance genes that are independent of selective pressures or recent transmission events. In addition, a high degree of taxonomic similarity across environments can provide clues to their potential ecological connectivity [60, 61]. Strong differences in biotic and abiotic conditions limit the ability of bacteria, and hence ARGs, to transfer and establish themselves in new environments, even if the physical distance is small, as illustrated, for example, by the differences in both taxa and ARGs between human body sites. The opposite is probably the explanation to why human gastrointestinal samples and those of animal origin (of which many are gastrointestinal) have a relatively large overlap in terms of ARGs and taxa. It would seem reasonable to assume that wastewater/sludge would harbour ARGs and taxa similar to those found in human gut simply because human faeces largely end up in sewage. However, we found that the ARGs and taxonomic profiles in wastewater/sludge microbiota had limited similarity to the human gut microbiota, as also reported by previously [53, 62–64], and was also much more variable between samples. An important explanation behind the discrepancy between faeces and wastewater is likely the difference in oxygen availability. Another study of ours shows that the strongest shift between the bacterial communities of human gut and wastewater microbiota is the almost complete elimination of the obligate anaerobes that dominate the gut microbiota [53].

Based on metagenomic analysis of known MGEs, environmental bacteria seem to be better adapted to transfer genes than those thriving on or in our bodies. Within the human microbiome, class 1 integrases (*intI1)* and ISCR transposases such as ISCR2, ISCR5 and ISCR8 were common, whereas external environments harboured both greater diversity and relative abundances of MGEs. This was observed despite the fact that MGEs are studied at much greater depth in human pathogens, which in turn would be expected to bias estimates towards more MGEs in human-associated environments. Some of the integron-associated integrases and ISCR transposases found in environmental metagenomes are often also associated with ARGs in clinical isolates [65, 66], whereas others have, at least not yet, been associated with resistance. Nevertheless, the widespread distribution of MGEs across external environments suggests ample opportunities for external environments to contribute to the mobilization and further transmission of ARGs.

Across all metagenomes, less than 1.5 % of all detected ARGs were exclusively found in the human microbiome. On the contrary, 57.5 % of the ARGs were only detected in metagenomes from environmental samples. Even though the majority of the investigated ARGs have been initially found in pathogens, our analysis suggests that most of them are still relatively rare in the human microbiota. Environmental samples generally contained a wider distribution of resistance genes to a more diverse set of antibiotics classes. For example, the relative abundance of beta-lactam resistance genes was much larger in external environments than in human and animal microbiomes. This suggests that the external environment harbours many more varieties of resistance genes than the ones currently known from the clinic. Indeed, functional metagenomics has resulted in the discovery of many novel ARGs in external environments [12, 55, 67, 68]. This all fits well with an overall much higher taxonomic diversity of environmental microbial communities. In terms of consequences associated with the potential transfer of ARGs to human pathogens, we argue that unknown resistance genes are of greater concern than those already known to circulate among human-associated bacteria [22].

## Conclusions

We used databases on known genes to estimate the overall structure and diversity of antibiotic resistomes and taxa in deeply sequenced metagenomes across environments, including humans and animals. Most importantly, we described the potential for many external environments, such as environments subjected to pharmaceutical pollution, air and wastewater/sludge to serve as hotspots for resistance development and/or transmission of ARGs. In addition, our results indicate that these environments may play important roles in the mobilization of yet unknown ARGs and their further transmission to human pathogens. Taken together, to provide guidance for risk-reducing actions, we suggest strict regulatory measures of waste discharges from pharmaceutical industries and encourage more attention to air in the transmission of antibiotic resistance.

## Methods

### Datasets and metadata

We included 864 metagenomes in this study, all generated using shotgun sequencing by Illumina technology and with sequencing depth of over 10 million reads per metagenome to allow more accurate determinations of relative gene counts and detection of less common resistance genes, MGEs and taxa [69].

In total, 358 publicly available metagenomes (Additional file 5: Table S4) spanning a range of external environments including soil (*n* = 200), water (*n* = 45), sediment (*n* = 60), mine (*n* = 7), wastewater/sludge (*n* = 32) and a Beijing smog event (*n* = 14), as well as 145 animal-associated metagenomes, were retrieved from MG-RAST (http://metagenomics.anl.gov/) [70] on 8 February 2015. We excluded datasets from plant-associated environments as only two samples passed our selection criteria. In addition to the environmental metagenomes, 350 metagenomes covering five main human body sites including gastrointestinal tract (*n* = 100), oral (*n* = 100), skin (*n* = 50), airways (*n* = 50) and urogenital tract (*n* = 50), from healthy adults, were retrieved from the Human Microbiome Project repository (http://hmpdacc.org/) [71] on 16 August 2015 (Additional file 6: Table S5).

Since MG-RAST lacked any deeply sequenced metagenomes from environments subjected to antibiotic pollution, and this is an apparent risk environment [49], we also conducted shotgun metagenomic sequencing of 11 sediment samples collected from an Indian river and two lakes polluted by pharmaceutical production, to represent an external environment where direct antibiotic exposure is apparent (Additional file 7: Table S6). Finally, all metagenomes analysed in this study were categorized into 13 different environment types based on their metadata, covering a wide ecological versatility of external, animal and human body habitats.

The number of air samples (Beijing smog) was limited. We therefore searched for other, high depth Illumina shotgun metagenomic data in different public repositories but were unable to find any. Thus, to enable comparisons with other air environments, we also analysed much smaller metagenomic air datasets generated using 454 technology from US cities, representing both outdoor and indoor environments from homes, offices and hospitals in New York and San Diego [31]. When comparing these samples to the Beijing smog samples, all samples were down-sampled to 350,000 reads per metagenome.

### Metagenomic sequencing

Eleven sediment samples from an Indian river and two lakes polluted by wastewater from pharmaceutical production were prepared for metagenomic sequencing (see [45] and [47] for details about sampling sites and procedures). Genomic DNA was extracted from the sediment samples using the PowerSoil® DNA Isolation Kit (MO BIO, Carlsbad, CA, USA) according to the manufacturer’s instructions. DNA purity and concentrations were measured using a NanoDrop™ spectrophotometer (Thermo Scientific, Waltham, MA, USA). Extraction of sufficient amounts of high-quality DNA from the polluted sediments was a challenge, likely due to chemicals in the sediment material interfering with the DNA extraction process. Therefore, extracted DNA was amplified for seven out of 11 samples using the REPLI-g Mini Kit (Qiagen, Hilden, Germany), according to the manufacturer’s instructions. Metagenomic shotgun sequencing libraries (101 bp paired-end) were prepared using the TrueSeq DNA Kit for multiplexing and sequenced on the Illumina HiSeq2000 sequencing platform.

### Pre-processing of dataset

Seqtk (v1.0-r82-dirty; https://github.com/lh3/seqtk) [72], which uses the modified Mott’s trimming algorithm, was used with the default error threshold of 0.05 for trimming low-quality reads, maintaining reads with minimum sequence length of 75 bp from all metagenomes. A few metagenomes from MG-RAST were only available in FASTA format without quality information. For those metagenomes, reads that had more than 10 % ambiguous bases were discarded. After quality filtering, 9.2 Tb of sequence data were left for downstream analysis.

### Resistance gene analysis

As the main rationale of this study was to identify potential risks for transmission of ARGs, we studied only known horizontally transferrable ARGs. Therefore, sequences of antibiotic resistance proteins and markers of MGEs such as integron-associated integrases (*intI*) and ISCR transposases were retrieved from the Resqu database (version 1.1; http://www.1928diagnostics.com/resdb/) [73], containing 3018 non-redundant protein sequences (divided into 325 resistance gene types) reported to be horizontally transferred between at least two different bacterial species and conferring verified resistance phenotypes. For resistance genes to antibacterial biocides and metals, we only studied the mobile resistance genes that frequently occur on plasmids. Therefore, only plasmid-borne antibacterial biocide and metal resistance protein sequences were retrieved from the BacMet predicted database (version 1.1; http://bacmet.biomedicine.gu.se/) which contains 40,556 non-redundant protein sequences (out of which 9173 are found on plasmids correspond to 131 biocide/metal resistance gene types) corresponding to resistance genes towards 43 chemical classes including 23 metals and 58 antibacterial biocides [74]. Note that our approach only detected resistance genes and MGEs that were highly similar to the reference sequences in the databases. Furthermore, as metagenomics does not link the genes to its larger genetic context or host, it cannot be inferred that the genotype is directly reflected in a corresponding resistance phenotype.

The quality-filtered dataset of 864 metagenomes was subjected to similarity searches against the BacMet and Resqu databases using USEARCH (v8.0.1445) [75]. To retrieve only the best matches, the entire coverage of the query reads matched against a target gene with a sequence identity threshold of 90 % was set (options “-usearch_global -id 0.9 –maxaccepts 1 -threads 16”). To avoid bias due to sequence length variations of resistance genes in the databases, the gene abundances were calculated by counting the number of raw read matches to each resistance gene or MGE, followed by normalization by the length of the respective gene. Subsequently, the length-normalized values were further normalized to 16S rRNA gene abundances divided by the average length of the 16S rRNA gene to minimize variance caused by differential extraction and analytical efficiencies and differences in background bacterial abundances [47]. The number of 16S rRNA copies in a genome can vary, usually between 1 and 15 [76]. Depending on the composition of microbial communities, the average 16S rRNA copy number may vary as well, but likely less so than between genomes. Normalization using single-copy genes such as *rec*A, *rpo*B, *gap*A, *gyr*B, *rpo*A or *pyr*H has a potential to reduce the between-sample variability and more directly reflect the abundance of bacterial cells in a sample [77, 78]. Accordingly, such genes, singly or in combination, have been suggested as alternatives to 16S rRNA for normalizing gene abundances in metagenomes to the bacterial fraction [79, 80]. However, many recent metagenomics studies investigating relative ARG abundances, including the present, either normalize simply to the total number of reads [29, 30, 55, 81–83] or to 16S rRNA [53, 84–86]. Still, we foresee that the use of single-copy genes for normalization could become more widely adapted in the future and thereby further improve the estimation of the relative abundance of resistance genes in bacterial communities.

Diversity of resistance genes, MGEs and bacteria was calculated using subsamples of 10 million high-quality reads from each metagenome. Although this sequence depth allow stratification of gene diversity across environments, rare resistance genes are likely to remain undetected. To identify the resistance genes that were shared between different environment types, resistance gene or taxa sharing matrices were generated using a weighted-model (both richness and relative abundance were accounted for) by applying a threshold (detection limit) for a gene or taxa to be counted as present (n_raw-counts_/n_total-sequenes_ > N_lowest-raw-counts_). If the number of raw read counts of the resistance gene or taxa (n_raw-counts_) in an environment, relative to total sequences in the environment type (n_total-sequences_), was higher than the lowest non-zero relative abundance (N_lowest-raw-counts_) in the environment type with lowest number of sequences present, the read count was included.

### Taxonomic affiliation

Reads corresponding to the 16S (SSU) rRNA genes were extracted from all the metagenomes using Metaxa2 (version 2.1) [87] for taxonomic assignment with default options. Taxonomic classification of the extracted bacterial 16S rRNA reads was carried out using the native Metaxa2 database of manually curated entries from SILVA (release 111) [88] and MITOZOA (version 2.0; release 10) [89]. This procedure assigned the reads matching to SSU rRNA to individual taxonomic levels up to species and/or subspecies level. The taxonomic raw counts of each metagenome were then normalized to counts per million reads.

### Statistical analysis

Beta-diversity was estimated based on metrics considering the presence/absence data (Sørensen’s (dis)similarity index) of resistance genes, MGEs and taxa (family level) according to the approach proposed in [32]). To further evaluate the ecological processes that drive high/low beta-diversity of resistance genes and taxa between samples, beta-diversity was additionally partitioned into ‘turnover’ (i.e. tendency to replace resistance genes or taxa with other genes or taxa, respectively) and ‘nestedness’ (i.e. the tendency to lose resistance genes or bacterial taxa, respectively) components, where larger numbers expressed larger diversity. Beta-diversity was computed using the ‘vegan’ statistical package [90] in R (http://www.r-project.org/) [91]. Heatmaps were generated in R using the ‘gplots’ package [92] to show the resistance genes and taxa that were most frequently found in different environments. Correlations between richness of resistance genes and genera were calculated using Spearman’s rank correlation. Similarly, partial correlations between richness of ARGs and biocide/metal resistance genes were calculated, while controlling for the effect of taxonomic richness, using partial correlation in the R package ‘ppcor’ [93]. Principal component analysis (PCA) was performed on the log-transformed normalized abundance data in R using the ‘prcomp’ function and visualised using the statistical package ‘ggplot2’ [94].

## Additional files

Additional file 1: Figure S1. Heat map showing the most abundant antibiotic resistance genes across environments. **Figure S2.** Relative abundance and richness of antibiotic resistance genes (ARGs) (per 16S rRNA) in air samples. **Figure S3.** Distribution of antibiotic resistance genes and bacterial genera among three ecologically distinct compartments—humans, animals and external environments. **Figure S4.** Correlations between richness of resistance genes. **Figure S5.** Heat map showing abundance of different classes of mobile genetic elements (integron-associated integrases and ISCR transposases) across environments. **Figure S6.** Relative proportion of classified and unclassified bacteria across environments. **Figure S7.** Heat map showing the most abundant bacterial genera across environments. (PDF 217 kb)
Additional file 2: Table S1. Relative abundance of antibiotic resistance genes in air samples. (XLSX 68 kb)
Additional file 3: Table S2. Detected antibiotic resistance genes (ARGs) across environments. (XLSX 19 kb)
Additional file 4: Table S3. Beta-diversity analysis of resistance genes, MGEs and taxonomic compositions across environments. (XLSX 15 kb)
Additional file 5: Table S4. Metadata for samples collected from MG-RAST repository. (XLSX 55 kb)
Additional file 6: Table S5. Metadata for samples collected from Human Microbiome Project (HMP) repository. (XLSX 25 kb)
Additional file 7: Table S6. Metadata for samples collected from pharmaceutically polluted environments. (XLSX 12 kb)
Additional file 8: Source code for bioinformatics and statistical analysis. (TXT 21 kb)

## Abbreviations

ARGs: Antibiotic resistance genes
BMRGs: Biocide/metal resistance genes
ISCR: Insertion sequence common regions
MGE: Mobile genetic elements
PCA: Principal component analysis
SSU rRNA: Small subunit ribosomal RNA
