# Supplementary material for: The structure and diversity of human, animal and environmental resistomes
Source: Microbiome. 2016 Oct 7;4:54. doi: 10.1186/s40168-016-0199-5 (PMC5055678; doi:10.1186/s40168-016-0199-5)
Supplement: Additional file 1: Figure S1. — Heat map showing the most abundant antibiotic resistance genes across environments. Figure S2. Relative abundance and richness of antibiotic resistance genes (ARGs) (per 16S rRNA) in air samples. Figure S3. Distribution of antibiotic resistance genes and bacterial genera among three ecologically distinct compartments—humans, animals and external environments. Figure S4. Correlations between richness of resistance genes. Figure S5. Heat map showing abundance of different classes of mobile genetic elements (integron-associated integrases and ISCR transposases) across environments. Figure S6. Relative proportion of classified and unclassified bacteria across environments. Figure S7. Heat map showing the most abundant bacterial genera across environments. (PDF 217 kb) [file 40168_2016_199_MOESM1_ESM.pdf]

Additional file 1: Additional Figures

The structure and diversity of human, animal and environmental resistomes

Chandan Pal[1,2], Johan Bengtsson-Palme[1,2], Erik Kristiansson[2,3] and D. G. Joakim Larsson[1,2]

[1]Department of Infectious Diseases, Institute of Biomedicine, Sahlgrenska Academy, University of Gothenburg, SE-413 46, Gothenburg, Sweden

[2]Centre for Antibiotic Resistance Research (CARe) at University of Gothenburg, Gothenburg, Sweden

[3]Department of Mathematical Sciences, Chalmers University of Technology, SE-412 96, Gothenburg, Sweden

Correspondence to: D. G. Joakim Larsson (joakim.larsson@fysiologi.gu.se)

Additional file 1: Figure S1. Heat map showing the most abundant antibiotic resistance genes across environments. **Figure S2.** Relative abundance and richness of antibiotic resistance genes (ARGs) (per 16S rRNA) in air samples. **Figure 3**. Distribution of antibiotic resistance genes and bacterial genera among three ecologically distinct compartments – humans, animals and external environments. **Figure S4**. Correlations between richness of resistance genes. **Figure S5**. Heat map showing abundance of different classes of mobile genetic elements (Integron-associated integrases and ISCR transposases) across environments. **Figure S6.** Relative proportion of classified and unclassified bacteria across environments. **Figure S7.** Heat map showing the most abundant bacterial genera across environments.

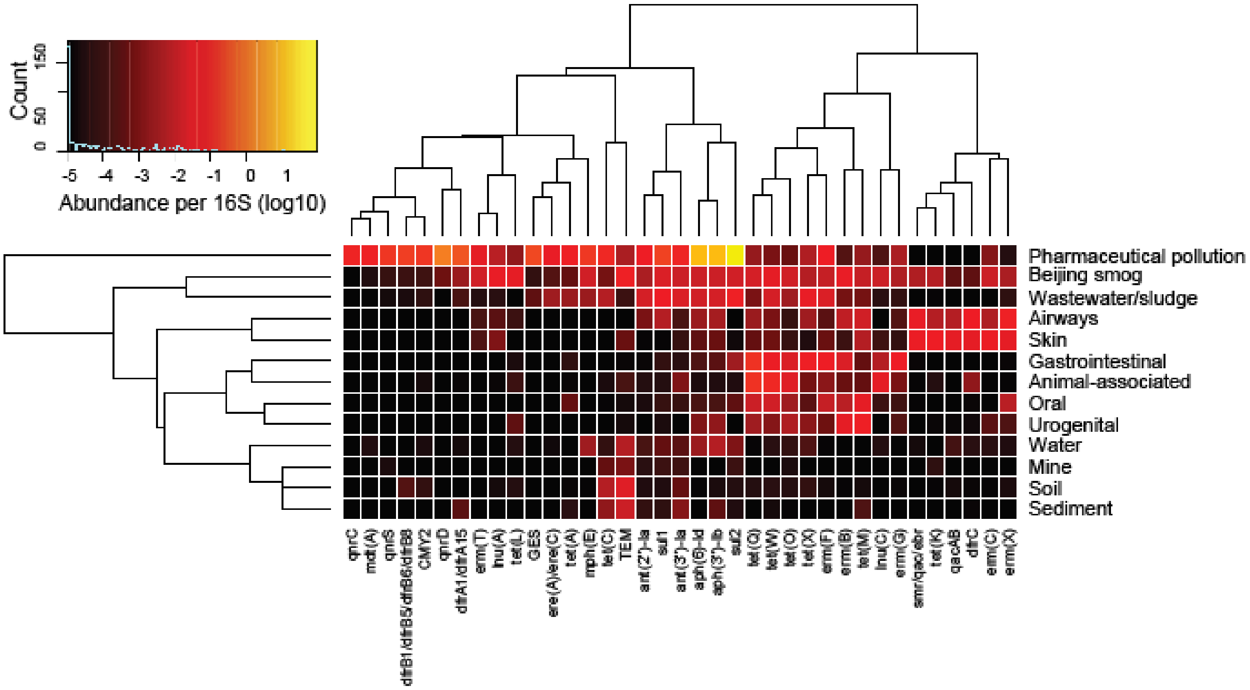

Figure S1. Heat map showing the most abundant antibiotic resistance genes across environments. The figure only shows the top 1% of the most abundant antibiotic resistance genes.

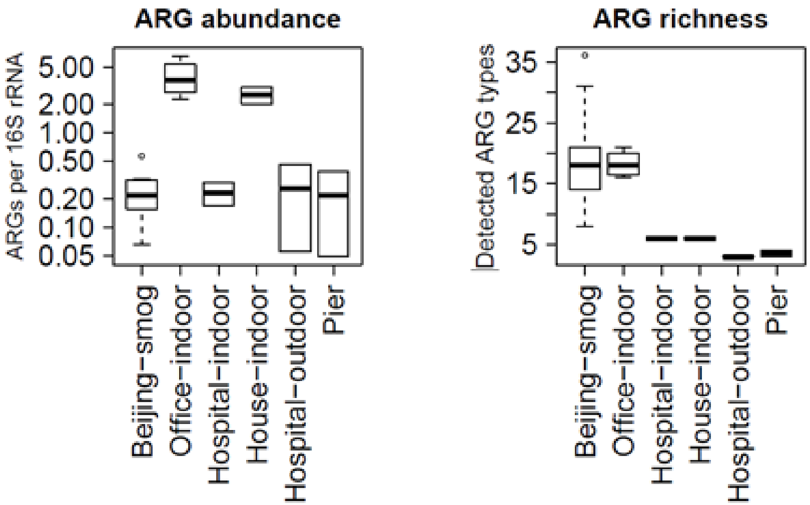

Figure S2. Relative abundance and richness of antibiotic resistance genes (ARGs) (per 16S rRNA) in air samples. To estimate the ARG richness, high-depth Illumina datasets from Beijing smog (average sequencing depth 75 million reads per sample) were down-sampled to 350,000 reads per sample and compared that with 454 datasets.

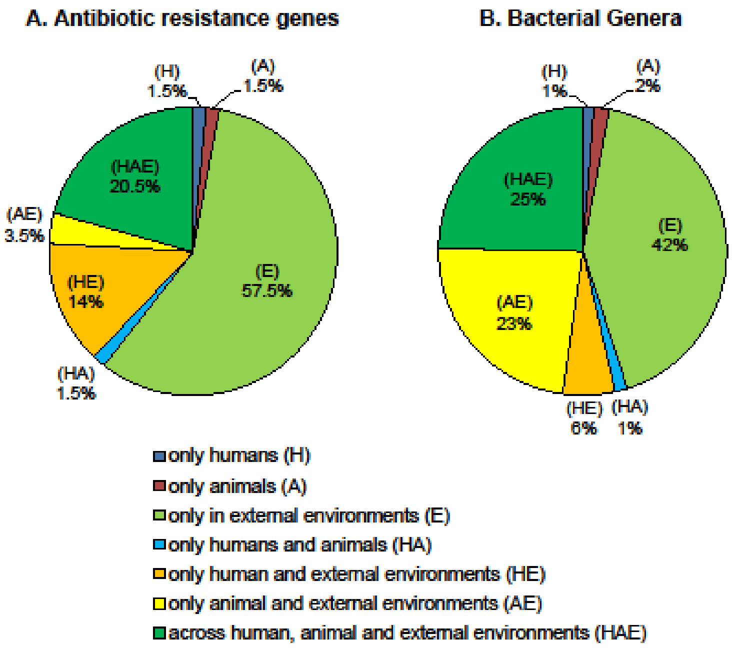

Figure 3. Distribution of **(A)** antibiotic resistance genes and **(B)** bacterial genera among three ecologically distinct compartments – humans, animals and external environments.

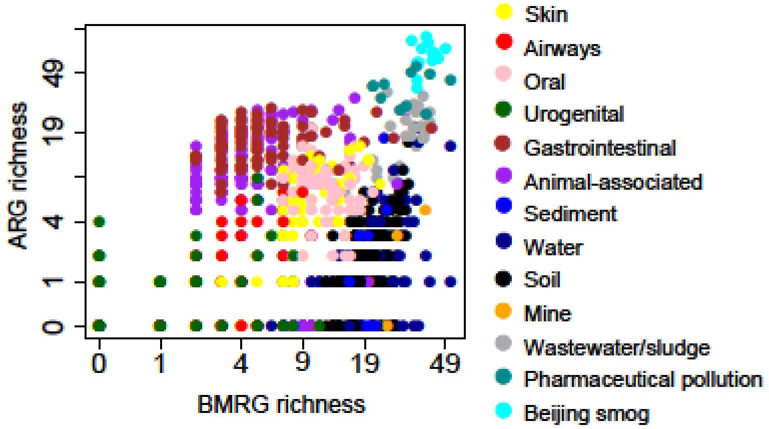

Figure S4. **Correlations between richness of resistance genes.** The figure showing the correlations between richness of biocide/metal resistance genes (BMRGs) and antibiotic resistance genes (ARGs) (Spearman's correlation coefficient=-0.015, p=0.645). Further, the correlation coefficient was calculated while the effect of taxonomic richness was controlled as 'covariate' (partial correlation coefficient=-0.056, p=0.0967). The richness values were log-transformed before performing Spearman's correlation, partial correlation and the statistical tests. A value of 1 was added to the richness matrix to avoid zeros in log-transformed richness values in the correlation test.

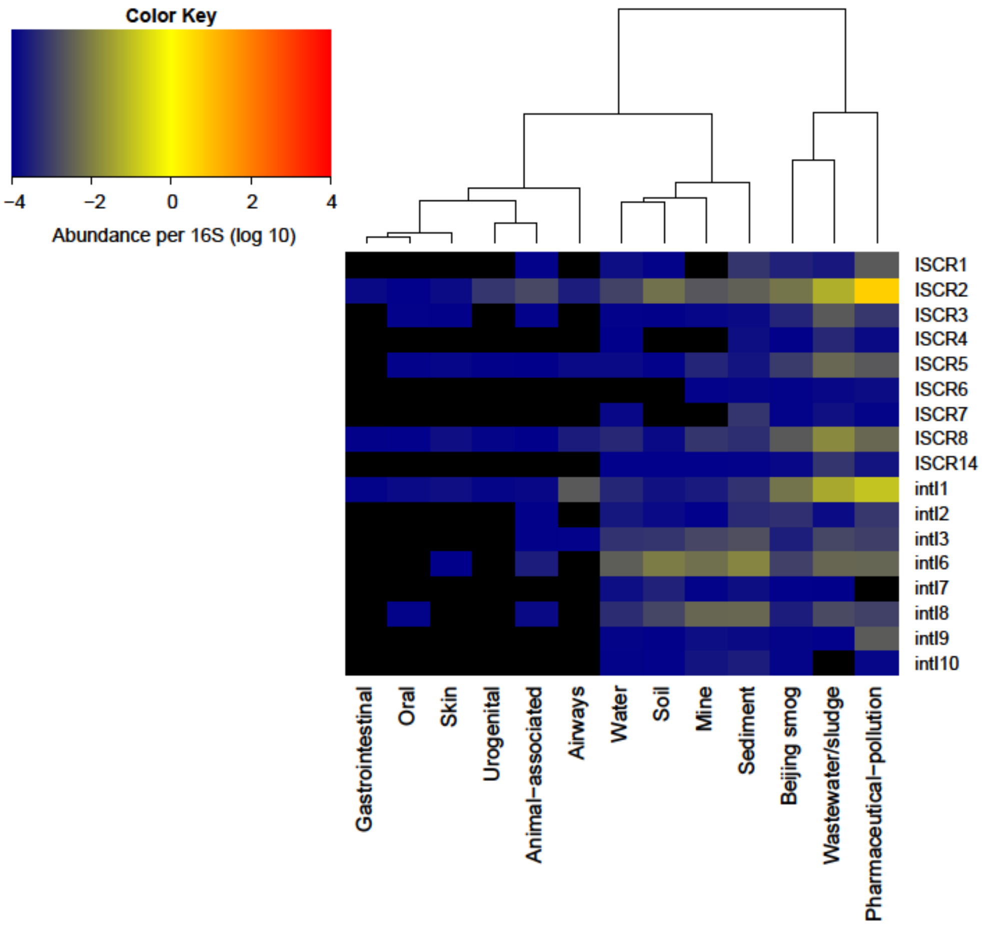

Figure S5. Heat map showing abundance of different classes of mobile genetic elements (Integron-associated integrases and ISCR transposases) across environments. Mobile genetic elements those have been found in over 0.0001 copies per 16S rRNA was counted as present in this heat map. MGEs found below that threshold in different environments are shown in black.

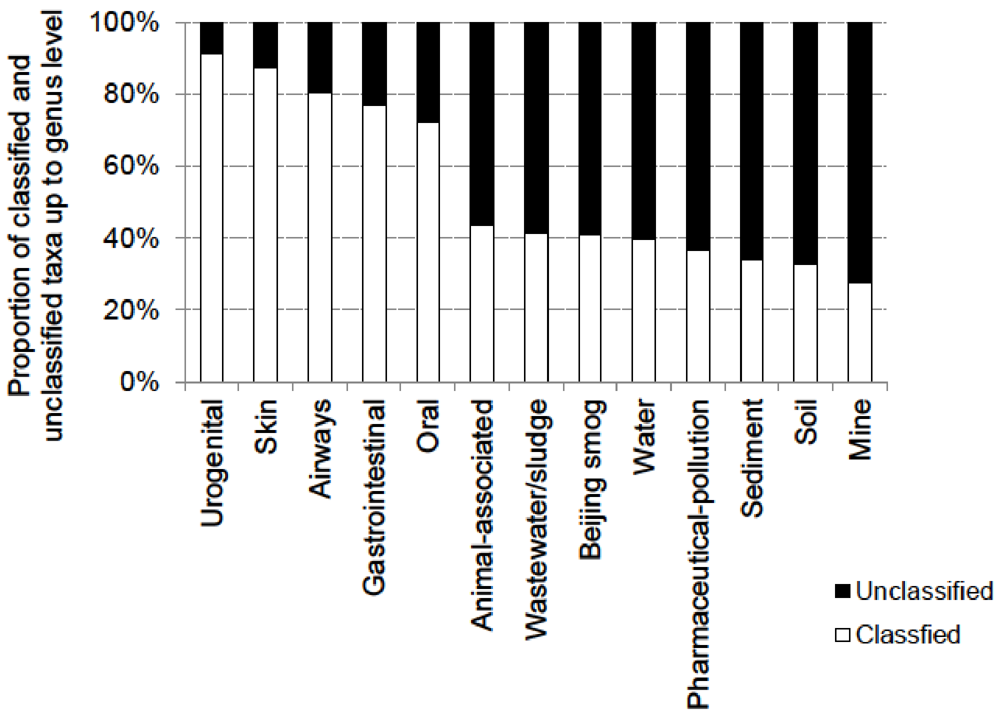

Figure S6. Relative proportion of classified and unclassified bacteria across environments. Bar graph showing the proportion of bacteria that have been successfully classified to genus level and the proportion of bacteria that have been unclassified up to genus level across all investigated environments including humans and animals.

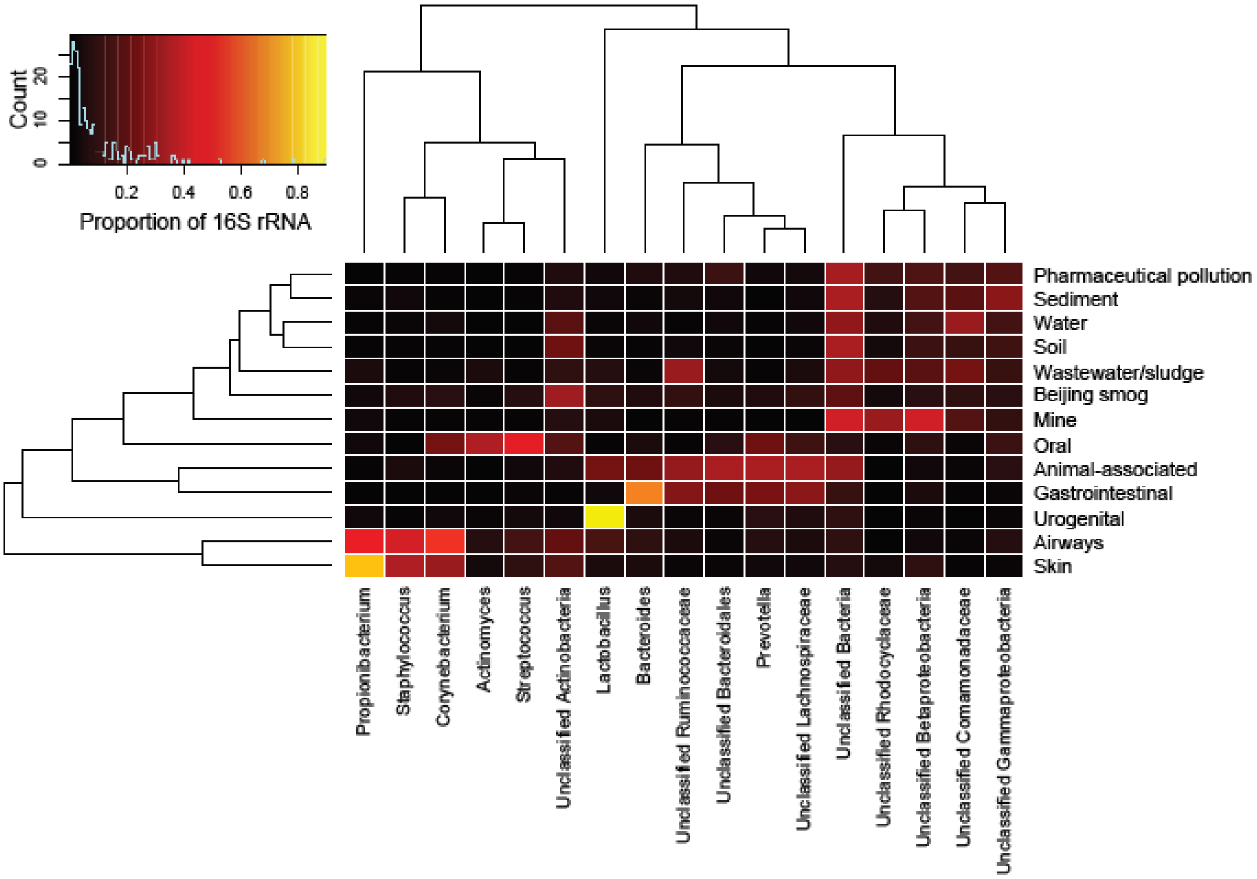

Figure S7. Heat map showing the most abundant bacterial genera across environments. Note that many bacteria were unclassifiable at different taxa level. The figure only shows the top 5% of the most abundant taxa.
